# Optimization of Hydrothermal and Diluted Acid Pretreatments of Tunisian* Luffa cylindrica* (L.) Fibers for 2G Bioethanol Production through the Cubic Central Composite Experimental Design CCD: Response Surface Methodology

**DOI:** 10.1155/2017/9524521

**Published:** 2017-01-24

**Authors:** Kaouther Zaafouri, Manel Ziadi, Aida Ben Hassen-Trabelsi, Sabrine Mekni, Balkiss Aïssi, Marwen Alaya, Latifa Bergaoui, Moktar Hamdi

**Affiliations:** ^1^Laboratory of Microbial Ecology and Technology (LETMi), The National Institute of Applied Sciences and Technology (INSAT), Carthage University, 2 Boulevard de la Terre, BP 676, 1080 Tunis, Tunisia; ^2^Department of Biotechnology and Environment Sciences, High Institute of Environmental Science and Technology (HIEST) of Borj-Cedria, Borj-Cedria Technopark, BP 1003, 2050 Hammam-Lif, Tunisia; ^3^Laboratory of Wind Energy Control and Waste Energy Recovery (LMEEVED), Research and Technology Center of Energy (CRTEn), Borj-Cedria Technopark, BP 95, 2050 Hammam-Lif, Tunisia; ^4^Laboratory of Materials Chemistry and Catalysis, Faculty of Sciences of Tunis, El Manar University, Tunis, Tunisia

## Abstract

This paper opens up a new issue dealing with* Luffa cylindrica* (LC) lignocellulosic biomass recovery in order to produce 2G bioethanol. LC fibers are composed of three principal fractions, namely, *α*-cellulose (45.80%  ± 1.3), hemicelluloses (20.76%  ± 0.3), and lignins (13.15%  ± 0.6). The optimization of LC fibers hydrothermal and diluted acid pretreatments duration and temperature were achieved through the cubic central composite experimental design CCD. The pretreatments optimization was monitored via the determination of reducing sugars. Then, the 2G bioethanol process feasibility was tested by means of three successive steps, namely, LC fibers hydrothermal pretreatment performed at 96°C during 54 minutes, enzymatic saccharification carried out by means of a commercial enzyme AP2, and the alcoholic fermentation fulfilled with* Saccharomyces cerevisiae*. LC fibers hydrothermal pretreatment liberated 33.55 g/kg of reducing sugars. Enzymatic hydrolysis allowed achieving 59.4 g/kg of reducing sugars. The conversion yield of reducing sugar to ethanol was 88.66%. After the distillation step, concentration of ethanol was 1.58% with a volumetric yield about 70%.

## 1. Introduction

The environmental crisis due to the increasing level of CO_2_ and the greenhouse gases emissions (GHG) in the atmosphere is linked to the global warming which is directly associated with the combustion of fossil fuels [1]. Consequently, to overcome these environmental and fossil energy issues, the development and utilization of alternative, nonpetroleum based renewable sources of energy became mandatory [1, 2]. Biomass and its byproducts, with a global production reaching 200 billion metric tons a year, represent great potential feedstocks for energy conversion technologies in order to produce biofuels [3]. Moreover, lignocellulosic biomass is renewable, more abundant, and the cheapest resource in the world. This biomass could be provided through food cultures (i.e., sugar cane, beets, corn, sorghum, and starch), energy or nonfood crops (i.e., switchgrass,* Miscanthus giganteus*, poplar, willow, sweet sorghum, wild sugarcane, bitter cassava, alfalfa, hemp, and water hyacinth), as well as from agricultural, forest and industrial residues (i.e., corn stover, sugarcane bagasse, rice straw [1], cassava pulp, palm residues, soybean residues, wheat straw, wheat bran [3], straw bark, used edible oil, and black liquor), woody feedstocks, softwood (pine and spruce), herbaceous biomass and cellulose wastes (waste office paper) [4], and seaweeds (brown algae) [5]. In the Mediterranean region, especially in Tunisia,* Luffa cylindrica* (LC) is a promising lignocellulosic feedstock for 2G bioethanol recovery [1]. LC is an annual herbaceous plant from the cucurbitaceous family [6]. It is a fibrous plant largely distributed in the tropical and subtropical countries and countries with moderate climate [7], with a plant growth yield reaching 62000 LC fruits/ha (20 to 25 fruits/plant). However, this yield depends highly on the climate [8]. Many common end-uses of LC fibers were listed as follows: the disposal of copper from food industry wastewater [9]; the biofilm supporting medium in trickling filters for wastewater treatment; the handicraft activities; some other industrial applications and pharmacology [10]; the bath sponges manufacturing; the use as a basic stamp for the chemical and biological immobilization and/or as a support with fixed bed of biological culture or for chemical synthesis; the use as a support of discoloration of the reagents and/or as a thermal support; and the use as a basic material for the insulation and the extraction of the chemical and biological compounds [6]. As listed previously and to the best of our knowledge, no published report exists on LC fibers recovery for 2G bioethanol production. In fact, the works dealing with this subject are still unknown and/or unwell and thorough studies are lacking. Thus, this work is considered as a novelty in terms of 2G bioethanol production from LC biomass. The second generation biofuels produced from renewable resources “plant biomass” are made with the lignocellulosic biomass since it is a cheap and abundant nonfood material available from plants [1]. Lignocellulosic substrate is mainly composed of two types of carbohydrates and one more complex polymer, namely, 30–55% of cellulose, 20–40% of hemicelluloses, 10–35% of lignins, and their ratio varies extremely depending on the plant species. These units are strongly linked and chemically bonded; in fact cellulose is the backbone structure, while hemicelluloses and lignins are the binding networks. Cellulose (consisting of D-glucose only) and hemicelluloses which is composed of mainly pentoses (like xylose and arabinose) and hexoses (like mannose, glucose, galactose, etc.) are bioconvertible [11, 12].

Three main steps are required to obtain 2G bioethanol from lignocellulosic biomass, namely, pretreatment, enzymatic saccharification, and fermentation and distillation [2, 11, 13, 14]. Many pretreatment methods of lignocellulosic biomass are listed in the literature, including physical pretreatment (grinding, milling, microwave, and extrusion), chemical pretreatment (hydrothermal-aqua Solv [1], alkali [1, 2], acid, organosolv, ozonolysis and ionic liquid), physicochemical pretreatment (steam explosion, liquid hot water, ammonia fiber explosion AFEX, wet-oxidation, and CO_2_ explosion), and biological pretreatment (delignification of lignocellulosic substrate by Basidiomycota fungi) [15]. Pretreatment step plays three important roles, that is, lignins destruction, hydrolysis of hemicelluloses, and modification of cellulose, which will improve enzymatic hydrolysis [15]. Particularly, hydrothermal pretreatment of lignocellulosic material for the enhancement of biofuels 2G production becomes more and more important in the 21st century. Water under high pressure and temperature can get into the biomass, moisturize cellulose, enhance its accessible and susceptible surface area, and improve its accessibility to the hydrolytic enzymes; indeed it removed hemicelluloses and part of lignins. The main advantages of hydrothermal pretreatment are as follows: no addition of chemicals, no requirement of corrosion resistant materials for hydrolysis reactors, and no need for size reduction of biomass; it requires much lower need for chemicals for neutralization of the produced hydrolyzate and it produces lower amounts of neutralization residues compared to many processes [1].

However, pretreatment methods have some weaknesses limiting their applications, so combined pretreatment methods are recently developed to curb this challenge, by increasing efficiency of sugars liberation, decreasing the formation of inhibitors, and making the process time shorter. Thus bioethanol yield becomes higher and the process becomes more economical [1].

In order to destroy cellulose chains, the subsequent enzymatic hydrolysis is catalyzed by the synergistic action of four cellulase enzymes operating at 40–50°C and pH 4-5, namely, endo-1,4-*β*-glucanases, cellobiohydrolases, exo-1,4-*β*-glucanases—that will hydrolyze cellulose into cellobiose—and *β*-glucosidases that will hydrolyze cellobiose into glucose. Cellulolytic enzymes play a critical role in lignocellulose saccharification and bioconversion of pretreated lignocellulosic material that requires multiple enzyme activities. The monomeric sugars (glucose, galactose, mannose, xylose, and arabinose) released from enzymatic saccharification are converted into ethanol thanks to some microorganisms.* Saccharomyces cerevisiae* is the most used yeast for ethanol production from hexoses given that it is well-known for its resistance to low pH, high temperatures, high ethanol concentration, and various inhibitors. Otherwise, one amylolytic* Saccharomyces cerevisiae* strain was employed for bioethanol production from wheat bran [3]. Other yeasts could produce ethanol through hexoses recovery, mainly from xylose, for example,* Pichia stipitis*,* Candida shehatae, Kluyveromyces marxianus, *and* Pachysolen tannophilus*. Some bacteria could also ferment monomeric sugars to produce alcohols, such as* Zymomonas mobilis* and* Escherichia coli *[13]. Several types of fermentation processes have been tested, for example, batch, continuous, continuous with cell recycling, fed-batch, and repeated-batch culture designs [15]. In order to obtain a fuel grade or anhydrous ethanol, many distillation and dehydration processes are used [13]. Nevertheless, the scale-up of the whole lignocellulosic biomass conversion process is very expensive. In order to reduce the biofuels 2G production cost, suitable processes available were listed in the literature, namely, the implementation of simultaneous saccharification and fermentation SSF, which integrates enzymatic saccharification and ethanol fermentation in one system, saving both process and time cost [1, 2, 15], the using of a recombinant cellulase cocktail (RCC), which contains two cellobiohydrolases, an endoglucanase and a *β*-glucosidase with* S. cerevisiae* in SSF condition [3], and the continuous recycling of enzymes during production of lignocellulosic bioethanol by using high dry matter content and low enzymes dosage and so reducing the enzyme consumption and as a result reducing their cost [16].

Otherwise, the success key of the bioethanol 2G process is the optimization of the different production steps [17]. Response surface methodology (RSM) is an optimization methodology commonly employed. In this methodology, the interaction effects between factors on the response of an analytical system could be illustrated by a surface in three dimensions, called the response surface. Among the several RSM design classes, central composite design (CCD) is among the most popular methods due to its simple structure and efficiency [18].

Regarding the aforementioned problematics, the main goal of this study is to optimize the hydrothermal and diluted acid pretreatments of Tunisian* Luffa cylindrica *fibers for 2G bioethanol production through the cubic central composite experimental design CCD and RSM. The effects of the main influencing factors, which are temperature, reaction time, and H_2_SO_4_ concentration on sugars concentrations, are studied. Besides, the subsequent 2G bioethanol from LC fibers process feasibility is carried out by means of maximizing the enzymatic saccharification of the pretreated substrate and testing the alcoholic fermentation of biomass hydrolysate.

The new concern of the current work is to explain the mechanisms of both hydrothermal and diluted acid pretreatments of Tunisian* Luffa cylindrica *fibers for 2G bioethanol production and thus to highlight the beneficial effects of hydrothermal pretreatment in favor of 2G bioethanol process effectiveness in terms of 2-G fermentable sugars.

## 2. Materials and Methods

### 2.1. Raw Material: Sampling and Preprocessing

*Luffa cylindrica* fresh fruits used for this study were sampled from the region of Monastir that is located in the Tunisian Sahel (center-east coast of Tunisia) in January 2014. The samples were milled with a kitchen grinder. Then, they were stocked in glass bottles at 4°C, for both characterization analysis and subsequent experimental procedure. This preprocessing step is considered as a mechanical pretreatment made before diluted acid and hydrothermal pretreatments steps and enzymatic saccharification and fermentation of LC fibers.

### 2.2. Analytical Methods

#### 2.2.1. Proximate and Ultimate Analysis of LC Fibers

Proximate analysis of LC fibers was carried out by the measurement of the dry matter, volatile matter, and the ash content according to the protocols described by Boussarsar et al. (2009) [19]. Ultimate CHN analysis of LC fibers was achieved with Perkin Elmer 2400 CHN elemental analyzer, in rich oxygen atmosphere. The sulfur percentage was measured for the studied fibers, via Horiba Jobin Yvon elemental sulfur analyzer [20]. However, the oxygen content was calculated by difference as follows:

(1) $$\begin{array}{c} O\left(\;\%\;\right)=100-\left(\;C+H+N+S+ash\;\right). \end{array}$$

#### 2.2.2. Density of LC Fibers

The LC fibers density was measured according to the protocol described by Hamza et al. (2013) [21].

#### 2.2.3. pH of LC Milled Fibers

The pH of LC milled fibers was determined according to the method detailed by Mukherjee et al. (2011) [22].

#### 2.2.4. Lignocellulosic Characterization of LC Fibers

The lignocellulosic characterization including cellulose, hemicelluloses, and lignins of LC fibers has been fulfilled according to a gravimetric method employing specific chemical reagents, described by Sun et al. (2003) [23] with some modifications related to the initial sample size for simplicity and repeatability. At first, 10 grams of LC milled fibers is defatted by using toluene and ethanol mixture (2 v/v) during 6 hours at ambient temperature, to determine the lipids content. Secondly, for water-soluble polysaccharides extraction, the defatted LC fibers were treated with 200 mL of water at 80°C for 2 hours. After that, a simultaneous treatment with sodium hypochlorite and acetic acid at pH 4 during 2 hours at 75°C was applied to the collected solid fraction from the previous step in order to determine the lignins content. Then, the holocellulose fraction obtained from the previous acid treatment was purified with 600 mL of sodium hydroxide (10% weight/volume) for 10 hours at 20°C under stirring conditions, to extract and purify the *α*-cellulose. After filtration of the previous reaction mix, the collected liquid fraction was neutralized with HCl chlorhydric acid (6 M), until reaching a pH about 5.5 and then precipitated with 450 mL of ethanol (95° alcoholic degree). The obtained pellets were washed with ethanol (70° alcoholic degree) then dried in a ventilation oven at 50°C in order to obtain the hemicellulosic fraction. All the experiments related to the lignocellulosic characterization of LC fibers were carried out in triplicate.

#### 2.2.5. Thermogravimetric Analysis TG-DTG of LC Fibers

The TG/DTG analysis of LC fibers was carried out using Setaram thermogravimetric analyzer type labsys® thermo-balance. The operating conditions were as follows: inert atmosphere (N_2_ nitrogen gas flow), temperature varying from 30°C to 900°C, heating rate about 10°C/min, and initial sample weight of 6.6 mg. The data were taken and recorded every 1.1 seconds [24].

#### 2.2.6. Fourier Transform Infrared Spectroscopy (FTIR) of LC Fibers

Fourier transform infrared spectroscopy (FTIR) analysis was performed for LC fibers. The translucent pellets (5 mm Ø) were done by blending and pressing LC milled fibers with KBr powder (5 : 100 w/w). The FTIR spectra was recorded in absorbance mode at a spectral range of 4000 and 400 cm^−1^ with an accumulation of 15 scans using spectrophotometer type Perkin Elmer Spectrum BX®, equipped with a He-Ne laser and with detector MCT type broadband and high sensitivity. The spectra acquisition was made via spectrum v5.3.1 software. The bands identification was accomplished according to the data cited by Feng and Donghai [25].

#### 2.2.7. Total Sugars Determination

The total sugars concentration of the studied samples was performed according to Dubois et al.'s (1956) method [26] by adding a phenol solution (5% w/v) and concentrated sulfuric acid H_2_SO_4_ (96%–98% v/v). Then, the samples incubation was achieved in a boiling water bath at 100°C for 5 minutes. The absorbance of each sample was measured at a wavelength *λ* = 480 nm using a spectrophotometer UV-visible type Jenway®. The total sugars concentration of each sample was determined referring to the standard curve previously established with the same protocol detailed above.

#### 2.2.8. Reducing Sugars Determination

The reducing sugars concentration was measured referring to the method described by Miller (1959) [27] by mixing the studied sample with the 3-5,dinitrosalicylic acid DNS reagent prepared with the potassium sodium tartrate (KNaC_4_H_4_O_6_·4H_2_O) and the sodium hydroxide (NaOH). The reaction happened in a boiling water bath at 100°C for 15 minutes. After the reaction cooling, the absorbance of each sample was determined at a wavelength about 540 nm using a spectrophotometer UV-visible type Jenway. The reducing sugars concentration of each sample was measured according to the standard curve previously elaborated with Miller protocol described above.

#### 2.2.9. Ethanol Determination

The ethanol concentration of distilled samples resulting from fermentation step was determined through high-performance liquid chromatography using Agilent® equipment with inverse C18 column PRONTOSIL 120-5-C18-AQ 5.0Xm (250 mm × 4.0 mm) and sulfuric acid 1 mM as mobile phase, at 25°C with a flow of 0.3 mL/min (analysis time: 30 min and injection volume: 20 *μ*L) [28].

### 2.3. Experimental Methodology

#### 2.3.1. Optimization of Diluted and Hydrothermal Pretreatments of LC Crude Fibers

The cubic central composite experimental design (CCD), which is the most popular second-order designs, was adopted as detailed previously in the literature [29, 30] in order to optimize process parameters for LC fibers hydrothermal and diluted acid pretreatments. CCD is a very commonly used form of response surface methodology (RSM) in order to evaluate the interaction of possible influencing parameters on the appropriate response with a limited number of planned experiments [29, 30]. The three-level (−1,0, +1) operating factors (independent variables) and their respective coded levels for both LC fibers hydrothermal and diluted acid pretreatments are summarized in Table 1. The (+1) value is the highest level of the operating factor and (−1) value is its lowest level. The average of these two values is assigned to (0) which is the central level of the studied factor. The coded level of each factor was selected according to their direct influence on the lignocellulosic biomass pretreatment according to previous studies [15, 31, 32]. The selected response for LC fibers diluted acid pretreatment is solely the variation (Δ) of total sugars concentration to which the variation (Δ) of reducing sugars amount was added for LC fibers hydrothermal pretreatment.

The theoretical matrix of cubic central composite experimental design CCD for optimization of diluted acid and hydrothermal pretreatments of LC fibers showing runs in standard order are, respectively, illustrated by Tables 2 and 3. For both LC fibers hydrothermal and diluted acid pretreatments, the experiments are performed in Erlenmeyer flasks of 250 mL, loaded until 40% of their volume using suspension of LC milled fibers at 0.33% (w/w) of dry matter in static conditions. The experiments requiring the temperature about 120°C are carried out in autoclave type Labtech® model LAC-5040S at a pressure of 1.2 bar.

*Statistical Analysis and Mathematical Model*. NemrodW® software version 9901 was used for the statistical analysis of the output variables obtained for CCD experiments and for the regression coefficients calculation [33]. In order to explore the functional relationship between the operating factors (*X*) and the responses (*Y*), a second-order polynomial model was adopted. The coded mathematical equation of the studied model is expressed as follows:

(2) $$\begin{array}{c} Y=\beta 0+\overset{k}{\underset{i=1}{\sum }}\beta iXi+\overset{k}{\underset{i=1}{\sum }}\beta iiXi^{2}+\sum \underset{i<j}{\sum }\beta ijXiXj+\epsilon , \end{array}$$

where* Y* is the response, *β*0 is the model intercept coefficient, *Xi* and *Xj* are the operating factors (independent variables) (*i* and *j* range from 1 to *k*), *βj*, *βjj*, and *βij* are the interaction coefficients of linear, quadratic, and the second-order terms, respectively, *k* is the number of independent variables (*k* = 3 for diluted acid pretreatment and *k* = 2 for hydrothermal pretreatment), and *ϵ* is the error [29, 30].

The interactive effects of the factors were examined using response surface plots derived from the chosen model. The optimal conditions showing the best yields of total and reducing sugars from hydrothermal pretreatment were adopted for the subsequent experiments of enzymatic saccharification and fermentation.

#### 2.3.2. Enzymatic Saccharification of Pretreated LC Fibers

Firstly, pretreated LC fibers suspensions were neutralized using (1 N) NaOH solution to reach pH 4. Then, the enzymatic saccharification was carried out in 100 mL of total reaction mix volume of LC pretreated fibers, using separately two commercial enzymes: Sumizyme AP2 (pectinase, cellulase, and hemicellulase activities: 54000 unit/g) and Sumizyme SPC (pectinase and cellulase activities: 6,000 u/g−1.000 u/g) provided as a powder compacted in zipped plastic bags, by Shin Nihon Chemical Co., Ltd. (Japan). AP2 and SPC were, respectively, added at a rate about 0.2% (w/w) and 0.005% (w/w) relative to the dry matter content of the lignocellulosic substrate and they were previously dissolved in 1 mL of sodium acetate buffer solution (0.1 M) (pH 4.0 ± 0.2). The reaction time is about 1 hour at a temperature of 60°C. Finally, the enzymatic hydrolysis was stopped by increasing the temperature to 85°C for 15 minutes. The monitoring of enzymatic saccharification was fulfilled through reducing sugars measurement (as described in the Section 2.2.8.). For the subsequent fermentation step, the best enzymatic saccharification condition giving the maximum reducing sugars content was selected.

#### 2.3.3. Alcoholic Fermentation of LC Hydrolysates

A 250 mL Erlenmeyer flask containing 100 mL of LC hydrolysates was inoculated with 10% (v/v) of 12 hours old preculture (exponential growth phase) of commercial yeast strain* Saccharomyces cerevisiae *supplied by Rayen® food company (Béja-Tunisia), grown on Sabouraud broth. The fermentation was conducted at 30°C and pH 4.8 ± 0.2, during 24 hours at shaking conditions (250 rpm).

#### 2.3.4. Distillation

In order to increase the final ethanol concentration, the fermentation broth distillation was carried out at 78.5°C by using standard column for simple distillation.

## 3. Results and Discussion

### 3.1. Characterization of LC Lignocellulosic Biomass

#### 3.1.1. Physicochemical Characterization of LC Fibers

Ultimate and proximate analyses of LC fibers, as far as their lignocellulosic composition, including determination of pH, density, dry, and volatile matter contents, ash, lipids, polysaccharides, cellulose, hemicelluloses, and lignins percentages, are detailed in Table 4. As it can be seen, the pH value of LC fibers is about 4.75 ± 0.2, which is considered as acidic pH value suitable for subsequent thermochemical and biochemical conversion of LC biomass for bioenergy recovery. LC fibers have a density value around 0.93 ± 0.45, which is slightly lower than that of the same substrate (1.48) studied by Laidani et al. (2012) [6] and it is higher than some other lignocellulosic fibers studied by Hamza et al. (2013) [21], such as Alfa (0.672 ± 0.011), Rush (0.417 ± 0.010), Palm (0.578 ± 0.036), and Palm stipe (0.220 ± 0.055). Although LC fibers density is lower than those of some lignocellulosic substrates, for example, coconut (1.150), sisal (1.500), and banana (1.350) fibers [21], the density values of vegetal fibers are significantly lower than glass fibers (2.500) [21]. LC fibers are classified as wet lignocellulosic feedstocks, since their dry matter content is around 5.5 ± 0.33% with the same tendency of Algerian LC fibers having a dry matter content around 7.5% [6], which is significantly lower than those of some dry fibers, such as Alfa (92.58%), Rush (90.61%), Palm (92.72%), Palm stipe (91.38%) [21]. The volatile matter of Tunisian LC fibers is around 3.56 ± 1.3% with 2 ± 0.1% of ash. This value is considerably higher than Brazilian LC fibers ash content (0.7 ± 0.2%) studied by Siqueira et al. (2010) [7], relatively to the growing conditions variability (climate, soil nutrients, etc.) and plant biology. The ash content of the studied fibers is similar to some other annual and perennial plants, for example,* Parthenium argentatum* (2.0%), kenaf (*Hibiscus cannabinus*) (2.2%), cotton stalks (2.2%) [34], and corncob (1-2%) [12]. The lipids (ethanol-toluene extractives) content of LC fibers (12.44 ± 0.5%) are higher than those of wood, nonwood, and annual or perennial plants, which are ranging from 1.2% to 10.7% [34]. The ultimate analysis of the Tunisian LC fibers shows that carbon, hydrogen, sulfur, nitrogen, and oxygen contents are about 47.667%, 5.626%, 1.498%, 1.245%, and 41.964%, respectively. These previous findings are almost similar to the ultimate analysis of some lignocellulosic feedstocks, such as Brazilian [35] and Algerian [6] LC fibers, Poplar* Populus nigra* L., Fern* Pteris vittata* L., and cellulose pulps [36] with some differences due to the geographical conditions and plant physiology. The studied fibers are rich in *α*-cellulose (45.80 ± 1.3)% and in hemicelluloses (20.76 ± 0.3)% with a small amount of polysaccharides not exceeding 7.86% ± 0.1. Besides, their lignins content is about 13.15% ± 0.6. This lignocellulosic composition is slightly similar to those of Brazilian LC fibers (63–65.5% of *α*-cellulose), as well as to the fibers of Algerian LC core (45% of cellulose) [6, 7, 34], and also to those of some other lignocellulosic biomasses, having an *α*-cellulose content ranging from 39.23% to 55.9% [21, 33], such as Tunisian and Algerian Alfa (*Stipa tenacissima*) stems,* Posidonia oceanica* fibers, nonwood substrate (*Prosopis alba*, etc.), some other wood (*Pinus pinaster*), rush, and palm leaflets and stipe. Certainly, LC composition depends on various factors, such as species, variety, soil type, weather conditions, and plant age [35]. These previous results confirm that LC fibers are suitable for 2G bioethanol production.

#### 3.1.2. Fourier Transform Infrared (FTIR) Spectra of LC Fibers

**Figure 1:**
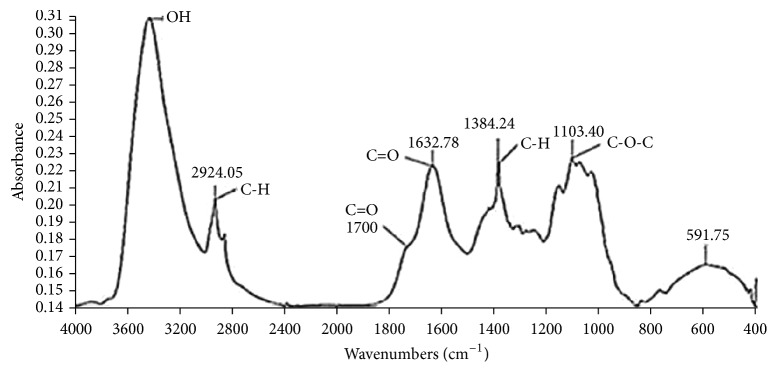
FTIR spectra of* Luffa cylindrica *crude fibers.

Figure 1 outlines the FTIR spectra of LC fibers. The bands assigned to the lignins are those around 3400 cm^−1^, 2924 cm^−1^, 1632 cm^−1^, and 1384 cm^−1^ which are, respectively, attributed to O-H stretching vibration, C-H stretching vibration, C=O stretching (unconjugated), and C-H bending vibration. The functional groups attributed to the hemicelluloses of LC are shown through three vibration bands existing around 1700 cm^−1^, 1384 cm^−1^, and 1103 cm^−1^, which are, respectively, attributed to ketone or aldehyde C=O stretching vibration, C-H bending vibration, and C-O-C asymmetrical stretching vibration. Cellulose fraction of LC fibers is emphasized by C-H bending vibration and C-O-C asymmetrical stretching vibration bands are detected, respectively, around 1384 cm^−1^ and 1103 cm^−1^. Consequently, these structural and functional characterizations of LC fibers confirm their aliphatic and oxygenated nature and thus their ability for bioethanol recovery as lignocellulosic feedstocks. These FTIR findings show the same tendency observed for some other lignocellulosic feedstocks, namely, Brazilian LC fibers [7, 35], Tunisian Alfa stem fibers [34], and rice straw [37].

#### 3.1.3. Thermogravimetric Analysis TG-DTG of LC Fibers

**Figure 2:**
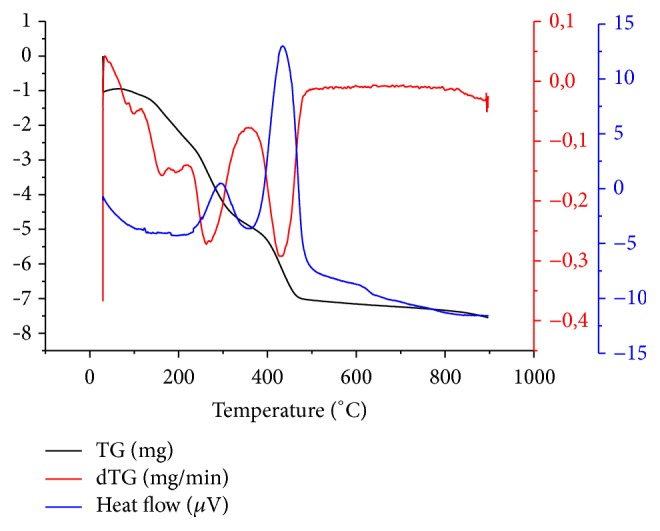
Thermogram DTA/TG/DTG of* Luffa cylindrica* crude fibers.

The thermal behaviour result of LC fibers is given in Figure 2. The black curve (TG) illustrates the mass loss (expressed in mg) of LC fibers, while the red curve gives the mass loss derivative (expressed in mg/min) and the blue curve presents the heat flow (expressed in *μ*V) applied during the thermal analysis. As shown in Figure 2, the thermal degradation curve of LC fibers shows three main decomposition stages. The first stage corresponds to the evaporation or drying process of the sample which happened from 30°C to 120°C with a slight weight loss about 3.8%, due to water removal and release of some light volatile molecules. This step is endothermic. The second stage of LC fibers thermal degradation generates a considerable weight loss about 53.0%, which is observed between 120°C and 360°C. This second event, which is exothermic, is related to the thermal degradation of hemicelluloses and cellulose occurring, respectively, at temperatures varying from 200°C to 350°C and from 350 to 400°C. This second region is considered as the main active pyrolytic stage. The maximum decomposition yield of LC fibers happens at 300°C given that the glycosidic linkage depolymerisation provokes the major weight loss [37]. Several previously studied lignocellulosic feedstocks show that the major mass loss rate is between 200 and 450°C, for example,* Posidonia oceanica *(L.) fibers (330°C) and sugar cane bagasse (395°C) and olive stones (380°C) [38] and rice straw (320°C) [37]. The third region corresponds to a continuous devolatilisation and lignins degradation occurring between 360°C and 510°C with a mass loss about 34.1% (exothermic stage). This third zone (400°C–700°C) is attributed to the passive pyrolysis zone [39]. From 510°C to 900°C, LC fibers thermal degradation progresses at a slow rate 7.6% because of the carbonaceous fraction decomposition of the residual solid sample. These findings confirm that the thermogravimetric analysis of LC fibers is in the right agreement with the thermal behaviour of the previous studied LC fibers [35, 40–42] and some other lignocellulosic biomass from different herbaceous plants [40–42].

### 3.2. Optimization of Diluted Acid and Hydrothermal Pretreatments of LC Fibers

The optimization of hydrothermal and diluted acid pretreatments process parameters of LC fibers were carried out by means of two cubic central composite experimental design (CCD) matrixes (as described above in the Section 2.3.1).

#### 3.2.1. Diluted Acid Pretreatment of LC Fibers

**Figure 3:**
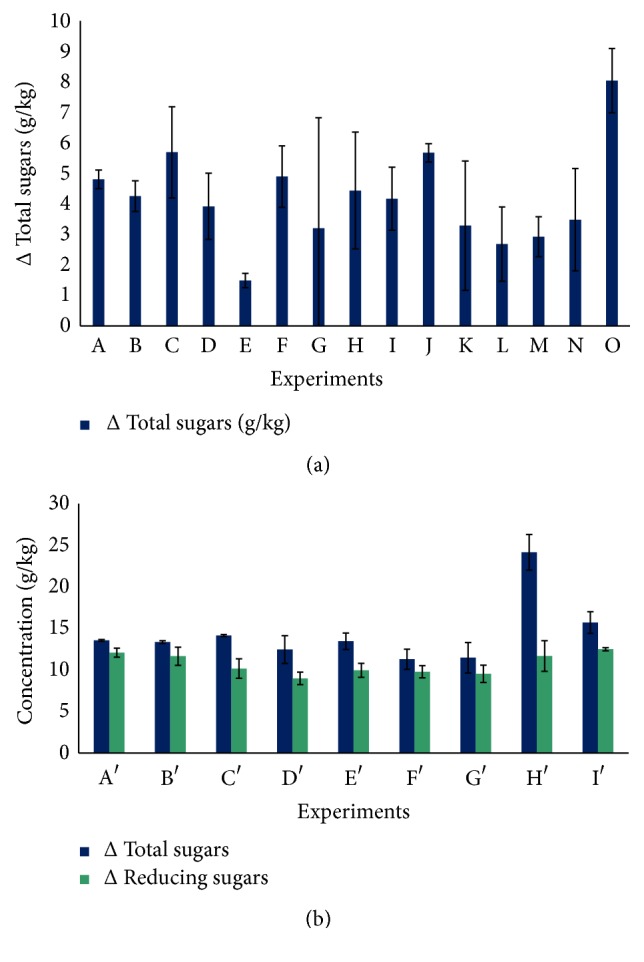
Variations Δ of sugars concentration for both diluted acid (a) and hydrothermal (b) pretreatments for all CCD experiments.

The studied response for LC fibers diluted acid pretreatment is the variation Δ of total sugars concentration. It is important to note that the variation Δ of reducing sugars concentration measurement for LC fibers diluted acid pretreatment demonstrates that the reducing sugars are absents.  Figure 3(a) illustrates the variation Δ of total sugars concentration during diluted acid pretreatment for all CCD experiments. From this figure, it can be seen that the optimum and the best variation Δ of total sugars concentration (8.039 ± 1.052) g/kg is obtained for the experience O [43–45] performed at 100°C during 45 minutes, with diluted H_2_SO_4_ at 2.75%. In order to conclude the effect of each factor influencing the diluted acid pretreatment of LC fibers, Table 5 summarizes the regression coefficients* C*_*T*_ for the variation Δ of total sugars concentration calculated by means of NemrodW software [33]. As is clear from Table 5, the regression coefficients* b*_1_ (+0.3821) and* b*_2_ (+0.1193) have positive signs, so both temperature and reaction time have positive effect on the variation Δ of total sugars concentration and they should be kept at their highest levels, respectively, at 120°C and 60 minutes. While the regression coefficient* b*_3_ has a negative sign (−0.4087), then H_2_SO_4_ concentration has a negative effect on the variation Δ of total sugars concentration. Consequently, the H_2_SO_4_ concentration should be used at 0.5%. The regression coefficients* b*_13_ (temperature *∗* acid concentration = +0.8708) and* b*_23_ (reaction time *∗* acid concentration = +0.0872) have positive effects on the variation Δ of total sugars concentration, whereas the interaction between temperature and reaction time (*b*_12_ = −0.4230) has a negative effect on the variation Δ of total sugars concentration. Besides, only the temperature increasing (*b*_11_ = +0.9075) has a positive effect on the variation Δ of total sugars concentration, which is the opposite for both reaction time (*b*_22_ = −1.0283) and H_2_SO_4_ concentration (*b*_33_ = −0.8120) effects.

**Figure 4:**
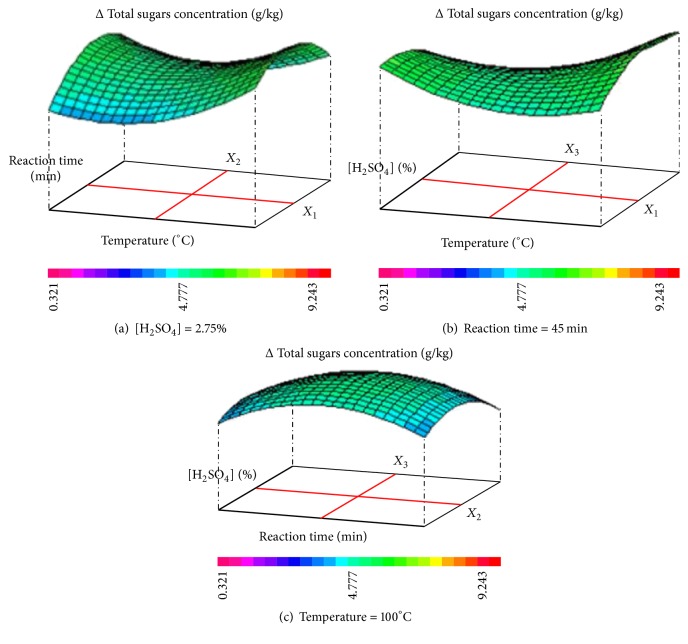
Responses surfaces of temperature-reaction time (a); [H_2_SO_4_]-temperature (b); [H_2_SO_4_]-reaction time (c); interaction effects on the variation Δ of total sugars concentration for CCD.

The mathematical model describing the variation Δ of total sugars concentration is established according to the calculated regression coefficients* C*_*T*_ as follows:
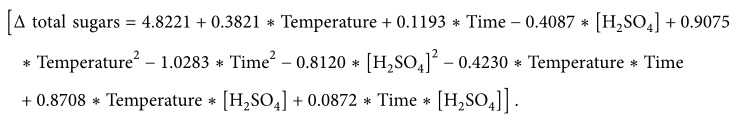
(3)
Figure 4 shows the responses surfaces of temperature-reaction time (a); [H_2_SO_4_]-temperature (b); [H_2_SO_4_]-reaction time (c); interaction effects on the variation Δ of total sugars concentration for CCD. As can be seen, the effect of interaction between different factors (temperature, reaction time, and H_2_SO_4_ concentration) on the variation Δ of total sugars concentration confirms that the optimal conditions of LC fibers diluted acid pretreatment are as follows: 100°C, 45 minutes, and H_2_SO_4_ at 2.75%, to reach Δ total sugars concentration around 8 g/kg. Theoretically, glucose is the main sugar present in LC's acid hydrolysis residue [7]. Choudhary et al. (2015) [43] confirm that the glucose concentration liberated, after sulfuric acid pretreatment of sorghum (YSS-10R variety), with 0.5% of H_2_SO_4_ at 100°C during 10 minutes, reaches 64 g/kg which is significantly higher than the optimal total sugars concentration released during this study. Otherwise, the acid thermal pretreatment of raw wheat bran was performed using 1% (w/v) of sulfuric acid in the autoclave at 121°C for 30 min [3].

The absence of reducing sugars released after LC diluted acid pretreatment could be explained by three main arguments, namely, (i) their degradation, (ii) their repolymerization or redistribution and/or, (iii) their transformation to the “enzymes and fermentation” toxic inhibitor compounds generated due to the thermo-acid conditions, for example, furfural, hydroxymethylfurfural HMF, levulinic, acetic, and formic acids, phenolics, and aldehyde components [13]. Consequently, avoiding the use of acid during the thermal pretreatment of LC fibers seems to be a better solution to preserve the structure of the reducing sugars.

#### 3.2.2. Hydrothermal Pretreatment of LC Fibers

The selected responses for LC fibers hydrothermal pretreatment are the variations Δ of both total and reducing sugars concentrations.  Figure 3(b) represents the variations Δ of both total and reducing sugars during hydrothermal pretreatment for all CCD experiments. As shown, the LC biomass hydrothermal pretreatment achieved at 100°C during 60 minutes (Experiment *H*′) allows reaching the optimal variation Δ of total sugars concentration about 24.161 ± 2.150g/kg. Otherwise, the optimal variation Δ of reducing sugars amount (12.490 ± 0.191 g/kg) is obtained for the experiment (*I*′) which was carried out at 100°C during 45 min. In order to study the influence of each factor affecting the hydrothermal pretreatment of LC fibers, Table 6 outlines the regression coefficients *C*_*T*′_ and* C*_*R*_ for, respectively, the variations Δ of total and reducing sugars concentrations for hydrothermal pretreatment of LC biomass, calculated using NemrodW software [33]. Table 6 indicates that the two* b*_0_ values (*C*_*T*′_ = −0.6586 and* C*_*R*_ = −0.2952) have negative signs so the raising of the temperature decreases both variations Δ of total and reducing sugars concentrations, although the reaction time increasing enhances the total sugars liberation given that* b*_2_ (*C*_*T*′_= +2.0452) has a positive sign. But the variation Δ of reducing sugars concentration decreases while the reaction time increases, since* b*_2_ (*C*_*R*_ = −0.4051) has a negative sign. Besides, the increase of the interaction between the temperature and the reaction time decreases both the variations Δ of total and reducing sugars concentrations, since* b*_12_ (*C*_*T*′_= −0.3982 and* C*_*R*_ = −0.1878) have negative signs.

**Figure 5:**
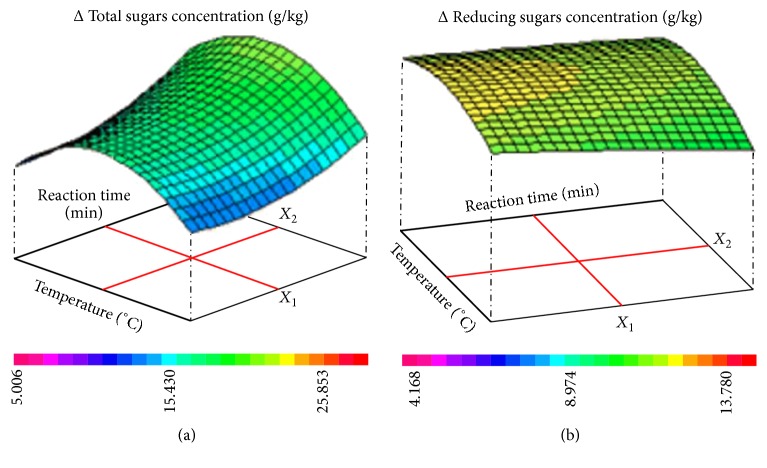
Responses surfaces of (temperature-reaction time) interaction effect on the variations Δ of total sugars (a) and Δ reducing sugars (b) concentrations for CCD.

The mathematical models describing the variations Δ of both total and reducing sugars concentrations are elaborated according to the calculated regression coefficients *C*_*T*′_ and* C*_*R*_ as follows:
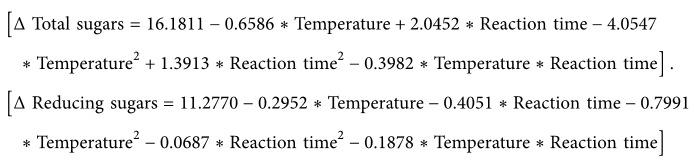
(4)
Figure 5 represents the responses surfaces of (temperature-reaction time) interaction effect on the variations Δ of both total sugars (a) and Δ reducing sugars (b) concentrations for CCD. As shown, the effect of interaction between the two main independent variables (temperature-reaction time) on the variation Δ of both total and reducing sugars concentrations demonstrates that the hydrothermal pretreatment of LC fibers performed at a temperature not exceeding 100°C during 45 minutes allows obtaining the variation Δ of total and reducing sugars concentrations, respectively, around 20 g/kg and 12 g/kg.

**Figure 6:**
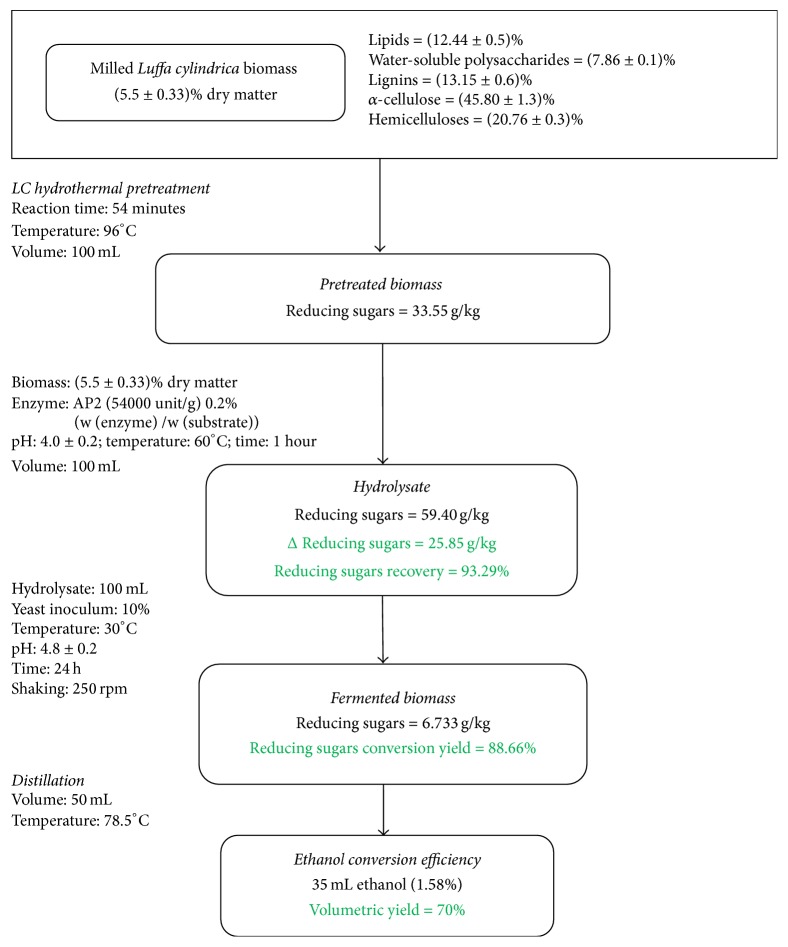
Process flowchart of* Luffa cylindrica* fibers pretreatment and enzymatic saccharification for 2G bioethanol conversion.

The reducing sugars concentration obtained after the hydrothermal pretreatment of LC fibers (33.55 g/kg) (Figure 6) is higher than the sugars amount (glucose plus xylose) released during thermal autoclaving pretreatments (121°C for 60 minutes) of wheat straw (14.8 ± 0.01 g/kg) used as substrate for biogas production [44]. Thus, LC fibers could be used as a potential source for bioethanol 2G recovery. Sánchez and Cardona (2008) [15] explain that the reducing sugars liberated after hydrothermal pretreatment (Liquid Hot Water LHW pretreatment method) are mainly obtained from hemicelluloses depolymerization and water-soluble polysaccharides hydrolysis. Besides, the hydrothermal pretreatment strength is the low or the absence of toxic inhibitors, which makes it one of the favorite pretreatment methods for the scientists to avoid inhibition of enzymatic saccharification and fermentation during bioethanol production.

Table 7 illustrates the desirability function of the variations Δ of both total and reducing sugars concentrations for CCD studying the optimization of hydrothermal pretreatment of LC biomass. As given by NemrodW software [33], the hydrothermal pretreatment performed in the optimal conditions should be fulfilled at a temperature about 95°C-96°C during 54–60 minutes. Furthermore, Chandra et al. (2012) found that the rice straw biomass hydrothermal pretreatment should be carried out for 10 min at 200°C [1].

### 3.3. Enzymatic Saccharification of LC Pretreated Fibers

The enzymatic saccharification of LC pretreated fibers was performed according to the protocol described above in Section 2.3.2. The highest reducing sugars concentration was about 59.4 g/kg, it records for the saccharification carried out by enzyme AP2. However, the enzymatic assay of LC pretreated fibers performed with enzyme SPC generates around 37 g/kg of reducing sugars.

The reducing sugars recovery after enzymatic saccharification performed with enzyme AP2 reaching 93.29% is higher than those of steam pretreated agricultural residues (triticale, Canadian prairie spring wheat (SW), durum wheat, feed barley, malt barley, oat, and flax straws) ranging from 30% to 70% [45]. Besides, the current finding is in perfect agreement with the maximum glucose yield around 85%, 89%, and 95%, respectively, obtained after enzymatic hydrolysis of hydrothermal pretreated switchgrass fibers, dilute acid pretreated cotton fibers, and steam pretreated* Arundo donax *[32, 46, 47]. Moreover, the current reducing sugars amount released after the enzymatic saccharification of LC hydrothermal pretreated fibers with the commercial enzymes AP2 and SPC (59.4 g/kg or 55.242 g/l and 37 g/kg or 34.41 g/l) is significantly higher than the reducing sugars liberated during SSF of alkali pretreated (80°C, 0.5 M NaOH/g dry weight for 2 h with agitation) sugarcane bagasse (12.468 g/l) aiming at the sugars and *β*-glucosidase production during single and mixed culture by* Trichoderma reesei* and* Penicillium decumbens* for 72 h [2].

### 3.4. Alcoholic Fermentation of Hydrolysate and Distillation

The alcoholic fermentation feasibility of AP2 hydrolysate giving the best reducing sugars rate (Δ reducing sugars = 25.85 g/kg) and the alcoholic broth distillation were carried out referring to the methodology detailed above, respectively, in Sections 2.3.3 and 2.3.4. After enzymatic saccharification, the reducing sugars conversion yield is around 88.66%. Thus, the ethanol conversion efficiency is 1.58% and its volumetric yield 70% which is higher than the ethanol conversion efficiency obtained from hydrothermal pretreated and enzymatic hydrolysate lucerne, ranging from 41.7% and 62.8% [48]. This amount is also more important than those reported for the alkali pretreated sugarcane bagasse fermentation, which is about 40.84% (of theoretical yield) achieved after 24 h using* Saccharomyces cerevisiae* [2] and for other lignocellulosic feedstocks, that is, dried carob pod particles fermented with* Zymomonas mobilis *(43%) [14] and microwave hydrothermal pretreated (900 W for 2 min) sago pith waste fermented with* Saccharomyces cerevisiae* (15.6%) [49]. However, the hydrothermal pretreated LC fibers ethanol conversion yield is slightly lower than the ethanol efficiency of wheat bran's starch fermented with* S. cerevisiae* yeast (81%–89%) [3]. To have a general overview of the studied biofuel refinery, Figure 6 outlines the process flowchart of LC fibers pretreatment and enzymatic saccharification for bioethanol 2G conversion.

## 4. Conclusion

From this research, it was highlighted that the hydrothermal pretreatment of LC fibers performed at 96°C during 54 minutes seems to be the suitable way to liberate the optimal amount of reducing sugars (33.55 g/kg). Then, the enzymatic saccharification was carried out via AP2 enzyme, and the reducing sugars concentration reached 59.4 g/kg. Then, 88.66% of reducing sugars were converted to alcohol. The potential conversion yield of 2G bioethanol is 1 Ton (dry matter) of LC fibers to 13.8545 kg (=3.6599 Gallon) of biofuel. Thus the current study approves that* Luffa cylindrica* could be considered as an energy crop for 2G bioethanol production in North Africa, especially in Tunisia.

## Figures and Tables

**Table 1:** The coded levels of the studied factors for both LC fibers hydrothermal and diluted acid pretreatments.

Pretreatment	*Diluted acid pretreatment*		
	*Hydrothermal pretreatments*		Acid concentration (%)
Factors	Temperature (°C)	Reaction time (min)	
Coded levels			
**−1**	80	30	0.5
**0**	100	45	2.75
**+1**	120	60	5

**Table 2:** Theoretical matrix of cubic central composite experimental design CCD for optimization of diluted acid pretreatment of *Luffa cylindrica* fibers.

	Experiments	Temperature (°C)	Reaction time (min)	Acid concentration (%)
A	1	−1	−1	−1
	2	−1	−1	−1
	3	−1	−1	−1

B	4	+1	−1	−1
	5	+1	−1	−1
	6	+1	−1	−1

C	7	−1	+1	−1
	8	−1	+1	−1
	9	−1	+1	−1

D	10	+1	+1	−1
	11	+1	+1	−1
	12	+1	+1	−1

E	13	−1	−1	+1
	14	−1	−1	+1
	15	−1	−1	+1

F	16	+1	−1	+1
	17	+1	−1	+1
	18	+1	−1	+1

G	19	−1	+1	+1
	20	−1	+1	+1
	21	−1	+1	+1

H	22	+1	+1	+1
	23	+1	+1	+1
	24	+1	+1	+1

I	25	−1	0	0
	26	−1	0	0
	27	−1	0	0

J	28	+1	0	0
	29	+1	0	0
	30	+1	0	0

K	31	0	−1	0
	32	0	−1	0
	33	0	−1	0

L	34	0	+1	0
	35	0	+1	0
	36	0	+1	0

M	37	0	0	−1
	38	0	0	−1
	39	0	0	−1

N	40	0	0	+1
	41	0	0	+1
	42	0	0	+1

O	43	0	0	0
	44	0	0	0
	45	0	0	0

**Table 3:** Theoretical matrix of cubic central composite experimental design CCD for optimization of hydrothermal pretreatment of *Luffa cylindrica *fibers.

	Experiments	Temperature (°C)	Reaction time (min)
A′	1	−1	−1
	2	−1	−1
	3	−1	−1

B′	4	+1	−1
	5	+1	−1
	6	+1	−1

C′	7	−1	+1
	8	−1	+1
	9	−1	+1

D′	10	+1	+1
	11	+1	+1
	12	+1	+1

E′	13	−1	0
	14	−1	0
	15	−1	0

F′	16	+1	0
	17	+1	0
	18	+1	0

G′	19	0	−1
	20	0	−1
	21	0	−1

H′	22	0	+1
	23	0	+1
	24	0	+1

I′	25	0	0
	26	0	0
	27	0	0

**Table 4:** Physicochemical properties and proximate and ultimate analysis and lignocellulosic composition of *Luffa cylindrica* fibers.

*Physicochemical properties*	

pH	4.75 ± 0.2
Density	0.93 ± 0.45

*Proximate analysis (wt.%)*	

Dry matter	5.5 ± 0.33
Volatile matter	3.56 ± 1.3
Ash	2 ± 0.1
Lipids	12.44 ± 0.5

*Ultimate analysis (wt.%)*	

H	5.626 ± 0.3
C	47.667 ± 1
S	1.498 ± 0.1
N	1.245 ± 0.1
O	41.964

*Lignocellulosic composition (wt.%)*	

Water-soluble polysaccharides	7.86 ± 0.1
Lignins	13.15 ± 0.6
*α*-cellulose	45.80 ± 1.3
Hemicelluloses	20.76 ± 0.3

**Table 5:** The regression coefficients *C*_*T*_ for Δ total sugars concentration calculated for diluted acid pretreatment of *Luffa cylindrica* biomass.

Regression coefficients	Factors and interactions	*C*_*T*_
*b*_0_	Squared effect term	4.8221
*b*_1_	Temperature	0.3821
*b*_2_	Reaction time	0.1193
*b*_3_	Acid concentration	−0.4087
*b*_11_	Temperature^2^	0.9075
*b*_22_	Reaction time^2^	−1.0283
*b*_33_	Acid concentration^2^	−0.8120
*b*_12_	Temperature*∗*Reaction time	−0.4230
*b*_13_	Temperature*∗*Acid concentration	0.8708
*b*_23_	Reaction time*∗*Acid concentration	0.0872

**Table 6:** The regression coefficients *C*_*T*′_ and *C*_*R*_ for, respectively, Δ total and reducing sugars concentrations calculated for hydrothermal pretreatment of *Luffa cylindrica* biomass.

Regression coefficients	Factors and interactions	*C*_*T*′_	*C*_*R*_
*b*_0_	Squared effect term	16.1811	11.2770
*b*_1_	Temperature	−0.6586	−0.2952
*b*_2_	Reaction time	2.0452	−0.4051
*b*_11_	Temperature^2^	−4.0547	−0.7991
*b*_22_	Reaction time^2^	1.3913	−0.0687
*b*_12_	Temperature*∗*Reaction time	−0.3982	−0.1878

**Table 7:** Desirability function of the variations Δ of the total and Δ reducing sugars concentrations for CCD studying the optimization of hydrothermal pretreatment of *Luffa cylindrica* biomass.

Parameters	Δ Total sugars concentration	Δ Reducing sugars concentration
Temperature (°C)	96	95
	(*X*_1_ = −0.216062)	(*X*_1_ = −0.260133)
Time (min)	60	60
	(*X*_2_ = 0.976316)	(*X*_2_ = 0.990424)
*Y*_1_ (Δ Total sugars) (g/kg)	19.541	19.571
*Y*_2_ (Δ Reducing sugars) (g/kg)	10.882	10.880
Desirability (*Y*_1_) (%)	51.70	52.39
Desirability (*Y*_2_) (%)	55.15	48.31
Desirability (%)	53.96	50.70

## Nomenclature

LC: Luffa cylindrica
SSF: Simultaneous saccharification and fermentation
CCD: Cubic central composite experimental design
RSM: Response surface methodology
AP2: Commercial cellulolytic enzyme
SPC: Commercial cellulolytic enzyme
FTIR: Fourier transform infrared spectroscopy
Δ: Variation
*C*_*T*_: The regression coefficients for Δ total sugars concentration calculated for diluted acid pretreatment of* Luffa cylindrica* biomass
*C*_*T*′_ and *C*_*R*_: The regression coefficients for, respectively, Δ total and reducing sugars concentrations calculated for hydrothermal pretreatment of* Luffa cylindrica* biomass.

## Additional Points

*Highlights*. (1)* Luffa cylindrica* hydrothermal pretreatment is performed at 96°C for 54 minutes. (2) The optimal reducing sugars amount obtained from pretreated biomass is 33.55 g/kg. (3) After enzymatic saccharification, reducing sugars recovery is 93.29%. (4) Reducing sugars conversion yield is 88.66% after alcoholic fermentation. (5) Bioethanol 2G conversion efficiency is 70%.
